# Rac1-mediated signaling plays a central role in secretion-dependent platelet aggregation in human blood stimulated by atherosclerotic plaque

**DOI:** 10.1186/1479-5876-8-128

**Published:** 2010-12-06

**Authors:** Suman Dwivedi, Dharmendra Pandey, Anna L Khandoga, Richard Brandl, Wolfgang Siess

**Affiliations:** 1 Institute for Prevention of Cardiovascular Diseases, University of Munich, Munich, Germany; 2 Department of Vascular Surgery, Clinic Schwabing, Munich, Germany; 3 Max-Planck Institute of Biochemistry, Martinsried, Germany

## Abstract

**Background:**

Platelet activation requires rapid remodeling of the actin cytoskeleton which is regulated by small GTP-binding proteins. By using the Rac1-specific inhibitor NSC23766, we have recently found that Rac1 is a central component of a signaling pathway that regulates dephosphorylation and activation of the actin-dynamising protein cofilin, dense and α-granule secretion, and subsequent aggregation of thrombin-stimulated washed platelets.

**Objectives:**

To study whether NSC23766 inhibits stimulus-induced platelet secretion and aggregation in blood.

**Methods:**

Human platelet aggregation and ATP-secretion were measured in hirudin-anticoagulated blood and platelet-rich plasma (PRP) by using multiple electrode aggregometry and the Lumi-aggregometer. Platelet P-selectin expression was quantified by flow cytometry.

**Results:**

NSC23766 (300 μM) inhibited TRAP-, collagen-, atherosclerotic plaque-, and ADP-induced platelet aggregation in blood by 95.1%, 93.4%, 92.6%, and 70%, respectively. The IC_50 _values for inhibition of TRAP-, collagen-, and atherosclerotic plaque-, were 50 ± 18 μM, 64 ± 35 μM, and 50 ± 30 μM NSC23766 (mean ± SD, *n *= 3-7), respectively. In blood containing RGDS to block integrin α_IIb_β_3_-mediated platelet aggregation, NSC23766 (300 μM) completely inhibited P-selectin expression and reduced ATP-secretion after TRAP and collagen stimulation by 73% and 85%, respectively. In ADP-stimulated PRP, NSC23766 almost completely inhibited P-selectin expression, in contrast to aspirin, which was ineffective. Moreover, NSC23766 (300 μM) decreased plaque-stimulated platelet adhesion/aggregate formation under arterial flow conditions (1500s^-1^) by 72%.

**Conclusions:**

Rac1-mediated signaling plays a central role in secretion-dependent platelet aggregation in blood stimulated by a wide array of platelet agonists including atherosclerotic plaque. By specifically inhibiting platelet secretion, the pharmacological targeting of Rac1 could be an interesting approach in the development of future antiplatelet drugs.

## Background

After rupture of atherosclerotic plaques thrombogenic matrix components and lipids are locally exposed to circulating platelets [1-5]. By adhering to these sites, platelets rapidly become activated, leading to secretion of their granule contents such as ADP that recruits circulating platelets into large aggregates culminating in the formation of platelet thrombi [5,6]. The latter are potentially life-threatening by occluding coronary and cerebral arteries.

The step-wise activation of platelets (adhesion, shape change, secretion and aggregation) involves an organized remodeling of the actin cytoskeleton. The major molecules involved in actin dynamics are the small GTP-binding proteins Rho, Rac, and Cdc42. These proteins differentially regulate the reorganization of the actin cytoskeleton, leading to the formation of different cellular structures. In platelets, Rho activation mainly regulates the Ca^2+^-independent cell spheration and contractility during shape change through stimulation of the Rho-kinase ROCK, whereas Rac1 has been reported to be essential for the formation of lamellipodia during platelet spreading [7-9]. Rac1 activation in platelets is Ca^2+^-dependent [10,11], and it has been shown to be involved in regulating secretion and subsequent aggregation in human platelets stimulated with thrombin [12,13]. However, in mice platelets, the results regarding the role of Rac1 in thrombin-induced aggregation and secretion are controversial [9,12,14]. By using conditional Rac1 knock-out mice, only one study showed impaired thrombin-induced aggregation [12]. In the two other studies, thrombin-induced secretion and aggregation were not affected; Rac1 was found to be involved only in collagen/glycoprotein VI-mediated platelet activation [9,14].

An important tool in studying the function of Rac1 is the compound NSC23766, a small-molecule inhibitor that fits into a surface groove of Rac1 known to be critical for the binding of specific guanine nucleotide exchange factors (GEFs) converting Rac-GDP into its active Rac-GTP form. NSC23766 inhibits i*n vitro *Rac1 binding and activation by the Rac-specific GEF Trio or Tiam1 [15]. The specific Rac-inhibitor NSC23766 has been used in more than 90 scientific studies in which the results obtained have often been validated by Rac-silencing and Rac knock-out experiments (see http://www.ncbi.nlm.nih.gov/pubmed).

By using NSC23766, our group recently unraveled a Ca^2+ ^-dependent pathway regulating secretion in thrombin-stimulated human platelets linking Rac1 activation to actin dynamics: Calcineurin→Rac1 →class-II PAKs activation→cofilin dephosphorylation and activation [13]. In the present study, we asked whether NSC23766 could inhibit human platelet secretion and aggregation induced by other platelet stimuli, particularly atherosclerotic plaque, and also whether it could reduce platelet function under more physiological conditions such as in blood. We report here that NSC23766 indeed blocks secretion and secretion-dependent aggregation in PRP and blood induced by ADP, TRAP, collagen and human atherosclerotic plaque, and notably plaque-stimulated platelet thrombi formation under arterial flow conditions. Such a broad inhibitory profile of a Rac1 inhibitor suggests that pharmacological targeting of Rac1 is an interesting approach for developing future antiplatelet drugs.

## Methods

### Materials

Acetylsalicylic acid was obtained from Fluka Chemie. Adenosine 3'-phosphate 5'-phosphate (ADP) was from Biopool (Wicklow, Ireland). Arg-Gly-Asp-Ser (RGDS) peptide was from Bachem Biochemica (Heidelberg, Germany). Albumin (fatty acid free) was purchased from Sigma. Collagen (Horm) was obtained from Nycomed Pharma (Unterschleißeim, Germany). Luciferase luciferin reagent was obtained from Chrono-Log corp (Havertown, PA). Microfluidic chambers were from Bioflux (Fluxion, San Francisco, California, USA). NSC23766 was obtained from Tocris Bioscience (Bristol, UK). Red blood cell (RBC) lysing buffer was from AbD Serotec (Oxford, UK).Formaldehyde was obtained from Sigma (Taufkirchen, Germany). Recombinant lepirudin was obtained from Pharmion (Refludan^®^, Germany). TRAP-6 (SFLLRN-OH, thrombin activating peptide) was from Bachem Biochemica (Heidelberg, Germany). The following monoclonal antibodies directly conjugated to fluorochromes were purchased from BD Biosciences (Heidelberg, Germany): phycoerythrin-(PE) conjugated anti-CD41a (HIP8) and fluorescein isothiocyanate-(FITC) conjugated anti CD62P (AK-4).

### Isolation of human atheromatous plaques

Atherosclerotic tissue specimens were collected from patients who underwent surgery for high grade carotid artery stenosis as described previously [16]. Patient consent was obtained and approved by the Ethics Committee of the Faculty of Medicine of the University of Munich. Plaque specimens were immediately frozen at -80°C after surgical removal. The atheromatous plaques, macroscopically visible by their yellowish color, were dissected under sterile conditions from other regions of atherosclerotic tissue. Calcified plaques were discarded. The plaques were characterized by histological analysis as atheroma with a thin fibrous capsule. Plaques were homogenized and processed as described [5,17]. The plaque concentration was adjusted to 100 mg/ml. Plaque homogenates from individual patients were pooled and used for the experiments.

### Preparation of blood

After informed consent was given, blood was collected from healthy volunteers using a 19-gauge needle and plastic syringe containing hirudin (~200U/ml in blood). In some of the experiments, acetylsalicylic acid (ASA) was added to the anticoagulant [17].The final concentration of ASA in the blood was 1 mM.

### Platelet aggregation and ATP-secretion in blood

Whole blood platelet aggregation was determined by impedance aggregometry as described previously [18]. In brief, a 1:1 mixture of 0.9% NaCl and whole blood was incubated for 5 min at 37°C whilst stirring in the presence or absence of different concentrations of NSC23766 and was then stimulated with collagen (0.5 μg/ml), atherosclerotic plaque homogenate (0.42 mg/ml), TRAP (5 μM) and ADP (5 μM). The increase in electrical impedance was recorded for 5 min, and the mean value of the area under the curve of two independent recordings (AU*min) was taken. For some experiments, blood with aspirin (1 mM) was taken and stimulated with ADP (5 μM) in the presence and absence of NSC23766 (300 μM).

For measuring ATP-secretion, a 1:1 mixture of 0.9% NaCl and whole blood was taken. The samples were pre-incubated with NSC23766 (300 μM) or solvent (water) for 5 min at 37°C whilst stirring (1000 rpm) in the aggregometer cuvettes. Luciferase-luciferin reagent (50 μl of 17.6 U/ml) was added for each reaction of 400 μl blood-saline mixture, and the increase of luminescence after exposure of stirred blood to platelet stimuli was recorded in the lumi-aggregometer (Chronolog, Havertown, PA)[19]. To some of the samples, RGDS (2 mM) or solvent (water) was added.

### Platelet aggregation and ATP-secretion in platelet rich plasma

Platelet-rich plasma (PRP) was prepared from hirudin-anticoagulated blood by centrifuging the blood at 160 × g for 20 min at room temperature (RT). Luciferin-luciferase was added, and aggregation of PRP and simultaneous ATP-secretion were determined at 37°C whilst stirring (1000 rpm) in the lumi-aggregometer. PRP whilst stirring was pre-incubated with different concentrations of NSC23766 or solvent (water) for 5 min at 37°C. In some of the samples, RGDS (1 mM) or solvent (water) was added 2 min before stimulation of PRP with ADP (5 μM), collagen (1.25 μg/ml), or atherosclerotic plaque homogenate (0.625 mg/ml). In some of the experiments, acetylsalicylic acid (1 M in ethanol) was added to the PRP (final concentration 1 mM) and incubated for 30 min. PRP was exposed to ADP (5 μM) in the presence or absence of NSC23766 (300 μM).

### P-selectin expression in PRP and blood

All experiments were performed in the presence of RGDS (1 mM). PRP (with and without aspirin pretreatment), stirred in the LABOR-aggregometer (Hamburg, Germany), was incubated with NSC23766 (300 μM) or solvent (water) for 5 min at 37°C before stimulation with collagen (5 μg/ml) or ADP (5 μM) for 2 min. Samples were fixed with equal volumes of Dulbecco's phosphate buffered saline (PBS) containing 3.7% formaldehyde for 30 min at room temperature. After fixation, samples were centrifuged in a microfuge for 5 min at 2300 × g. Pellets were washed twice with PBS. The pellets were incubated for 15 min in the dark at room temperature with CD62P-FITC or IgG- FITC (6 μl). P-selectin positive cells were quantified by flow cytometry (FACScan, Becton Dickinson, NJ, USA) and CELLQuest software. For each sample, a minimum of 10000 events was counted. For analysis, the percentage of positive cells was counted, and isotype matched IgG-FITC labeled platelets were subtracted from CD62P-FITC labeled platelets.

For P-selectin expression in blood, all experiments were performed in the presence of RGDS (2 mM). Aliquots (600 μl) of blood (0.9% NaCl and blood 1:1 mixture) were incubated with NSC23766 (300 μM) or solvent (water) for 5 min at 37°C whilst stirring in an impedance aggregometer (Multiplate^® ^analyzer, Dynabyte Medical; Munich) before stimulation with collagen (5 μg/ml) or TRAP (5 μM). After 2 min, an aliquot of 100 μl blood was added to 1.5 ml 1 × RBC lysis buffer, and platelets were fixed for 1 hour at room temperature. After fixation, samples were centrifuged in a microfuge for 8 min at 2300 × g. Pellets were washed twice with PBS. The pellets were incubated for 15 min in the dark at room temperature with CD41a-PE and CD62P-FITC (6 μl each). Platelets were gated by CD41a-PE fluorescence, and P-selectin positive cells were quantified by flow cytometry (FACScan, Becton Dickinson, NJ, USA) and CELLQuest software as described above.

### Analysis of platelet adhesion and thrombus formation in flowing whole blood

For flow experiments, T-BIO-FLUX200 (Fluxion, San Francisco, California, USA) with high shear plates (48 wells, up to 200dyne/cm^2^) was used. The microfluidic chambers were coated with 20 μl of plaque homogenate (5 mg/ml) dissolved in PBS containing 0.1% fatty acid-free albumin from the outlet channel. Care was taken to coat the viewing window of the channel and to leave the inlet channel free. The plaque coating was allowed to dry at room temperature overnight. Before the experiment, the channels were perfused with PBS (containing 0.3% albumin) for 10 min at a wall shear rate of 500s^-1^. Then hirudin-anticoagulated blood containing mepacrine (10 μM) in order to visualize platelets was added to the inlet well, and chambers were perfused for 10 min at a wall shear rate of 1500 s^-1^.

The plaque-coated microfluidic high shear plates were mounted on the stage of an upright microscope (Nikon TE2000E-PFS, Tokyo, Japan). Control blood and blood with NSC23766 (300 μM) was prewarmed to 37°C for 5 min prior to the start of flow, and experiments were performed at 37°C. Platelet deposition was observed and recorded in real-time (100 frames per sec) with a CCD camera (CooLSNAP HQ2, Tuscon AZ; USA). We used bright field and fluorescence microscopy for real-time visualization of platelet adhesion and aggregation in flowing blood. Control blood and blood containing NSC23766 were observed simultaneously in parallel channels. For each flow experiment, perfused surface fields of the size of 237900 μm^2 ^(located in the middle of the channels of the viewing window) were recorded, and fluorescence images were later analyzed off-stage by quantifying the area covered by platelets with the software NIS-element 3.0 version. In each field, the areas covered by platelets were quantified.

### Statistical analysis

Results are reported as mean ± SD from 3-7 experiments conducted with blood or PRP from different donors. Statistical significance was assessed by either paired Student's t-test or signed rank test where appropriate. Differences were considered significant when *p *was < 0.05.

## Results

### NSC23766 inhibits platelet aggregation upon stimulation of blood and PRP by TRAP, collagen and atherosclerotic plaque

**Figure 1:**
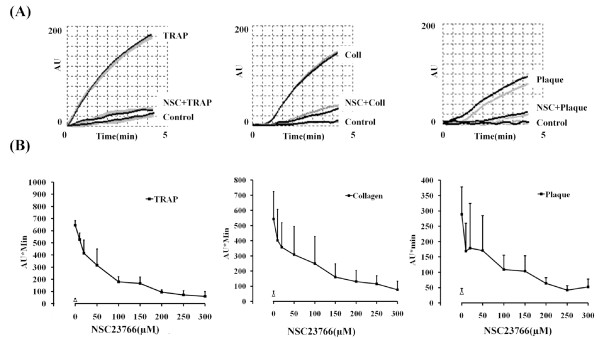
**Effect of NSC23766 on stimulus-induced platelet aggregation in blood**. (A) Hirudin-anticoagulated blood was pretreated with NSC23766 (300 μM) or solvent (H_2_O) for 5 min whilst stirring at 37°C before stimulation with TRAP (5 μM), collagen (0.5 μg/ml) or atherosclerotic plaque homogenate (0.62 mg/ml) for 5 min; representative impedance tracings. (B) Dose-response curves of NSC23766; values are mean ± SD (*n *= 4).

Platelet aggregation in blood induced by TRAP (5 μM) activating the PAR-1 receptor was reduced by 300 μM NSC23766 from 644 ± 37 to 59 ± 40 AU*min (control 29 ± 13 AU*min; *n *= 3) which corresponds to 95.1% inhibition (Figure 1). The IC_50 _of NSC23766 for inhibition of TRAP-stimulated aggregation was 50 ± 18 μM.

Platelet aggregation stimulated by collagen (0.5 μg/ml) was reduced by 300 μM NSC23766 from 542 ± 181 to 76 ± 56 AU*min (control 43 ± 25 AU*min; *n *= 7) which amounts to 93.4% inhibition of (Figure 1). The IC_50_ of NSC23766 for inhibition of collagen-stimulated aggregation in blood was 64 ± 35 μM.

 Plaques contain collagenous structures that directly stimulate platelet adhesion and aggregation which is mediated mainly by stimulation of GPVI [5]. Platelet aggregation induced by plaque was reduced by 300 μM NSC23766 from 289 ± 89 to 52 ± 26 AU*min (control 33 ± 13 AU*min; *n *= 3) which corresponds to 92.6% inhibition (Figure 1). The IC_50 _of NSC23766 for inhibition of plaque-stimulated aggregation in blood was found to be 50 ± 30 μM.

We also found that NSC23766 dose-dependently inhibited stimulus-induced aggregation of PRP (additional files 1 and 2, Figures S1 and S2). Platelet aggregation stimulated by collagen and plaque was completely inhibited by 300 μM NSC23766. The IC_50 _of NSC23766 for inhibition of collagen and plaque-stimulated aggregation of PRP was found to be 47 ± 14 μM, and 57.5 ± 20 μM, respectively.

### NSC23766 inhibits platelet ATP-secretion upon stimulation of blood and PRP by TRAP, collagen, and atherosclerotic plaque

**Figure 2:**
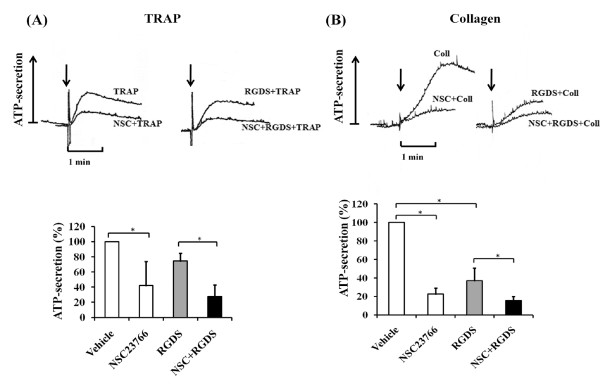
**Effect of NSC23766 on stimulus-induced ATP-secretion in blood**. Blood was pre-incubated with or without 300 μM NSC23766 (for 5 min), with or without 2 mM RGDS (for 2 min; added 3 min after NSC23766 or H_2_O) whilst stirring at 37°C before stimulation with (A) TRAP (5 μM) and (B) collagen (0.5 μg/ml). Top, tracings of ATP-secretion of blood. Bottom, bar diagrams; numbers are % of maximal ATP-secretion induced by TRAP (5 μM) and collagen (0.5 μg/ml), respectively. Values are mean ± SD (*n *= 3-4). * *p *< 0.05.

Inhibition of stimulus-induced platelet aggregation in blood by NSC23766 might be due to inhibition of secretion as observed previously in our study of thrombin-stimulated washed platelets [13]. Therefore, we studied the effect of NSC23766 on dense granule secretion by measuring the ATP-secretion in stirred blood. NSC23766 (300 μM) inhibited ATP-secretion induced by 5 μM TRAP (Figure 2A) and 0.5 μg/ml collagen (Figure 2B) by 60 ± 31% (*n *= 4) and 78 ± 7% (*n *= 6), respectively. In order to study the effect of NSC23766 on secretion independent of platelet aggregation, blood was pre-incubated with RGDS (2 mM) to block the integrin α_IIb_β_3_. RGDS reduced ATP-secretion by 26 ± 10% (*p *< 0.003; *n *= 4) in TRAP-stimulated blood and by 63 ± 14% (*p *< 0.04; *n *= 6) in collagen-stimulated blood (Figure 2A, B). Further pre-incubation with NSC23766 (300 μM) inhibited ATP-secretion by 73 ± 15%(*p*< 0.03 *n *= 4) and by 85 ± 4% (*p *< 0.004 *n *= 6) after stimulation with TRAP and collagen, respectively.

In PRP, RGDS reduced ATP-secretion by 92 ± 3% when stimulated with collagen and by 86 ± 7% when stimulated with plaque (additional files 1 and 2, Figure S1B, Figure S2B). Additional pre-incubation with NSC23766 (300 μM) inhibited ATP-secretion by 98 ± 1% in collagen-stimulated PRP (RGDS vs.RGDS+NSC23766: *p*< 0.03; *n *= 4) and by 99 ± 1% in plaque-stimulated PRP (*p*< 0.04 *n *= 4). The results in PRP support our findings in blood that NSC23766 inhibits platelet aggregation due to inhibition of secretion.

### NSC23766 inhibits ADP-induced aggregation of platelets in blood and PRP

**Figure 3:**
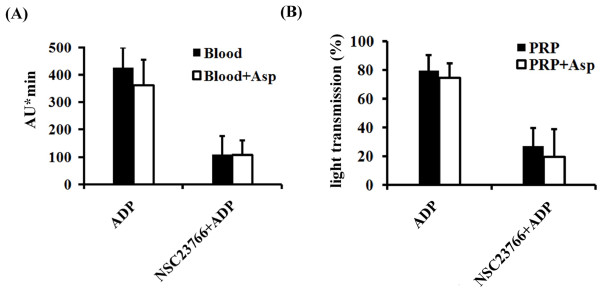
**Effect of NSC23766 on aggregation of platelets in blood and PRP stimulated with ADP**. (A) Blood (with or without aspirin) or (B) PRP (with or without aspirin) was pre-treated with 300 μM NSC23766 for 5 min whilst stirring at 37°C before stimulation with ADP (5 μM). Aggregation values of PRP are % of maximal aggregation induced by collagen (5 μg/ml). Values are mean ± SD (*n *= 4). * *p *< 0.05.

The extent of inhibition of stimulus-induced ATP-secretion in blood by NSC23766 (60-80%) was less than that of inhibition of platelet aggregation (92-95%). This discrepancy might be explained by an inhibitory action of NSC23766 on the platelet stimulatory effect of the remaining secreted ADP. Indeed, NSC23766 inhibited ADP-induced platelet aggregation in blood and PRP; this inhibition was 70% and 75%, respectively (Figure 3A, B).

### NSC23766 inhibits P-selectin expression on platelets upon stimulation of blood and PRP

To study whether NSC23766 also inhibits α-granule secretion, we examined the platelet surface expression of P-selectin in the presence and absence of NSC23766 in stirred blood containing RGDS. We found that NSC23766 completely inhibited P-selectin expression after stimulation with TRAP (5 μM) and collagen (5 μg/ml) (Table 1). Also in PRP, NSC23766 effectively inhibited P-selectin expression induced by ADP (5 μM) and collagen (5 μg/ml) (Table 2).

**Table 1:** Effect of NSC23766 on P-selectin expression of platelets in blood stimulated by TRAP and collagen

Agonist	P-selectin expression (% positive cells)	
	**Control**	**Stimulated**
	
TRAP (5 μM)	1.6 ± 0.6	6.8 ± 3.4
TRAP+NSC23766 (300 μM)		1.4 ± 0.6
Collagen (5 μg/ml)	1.7 ± 0.9	8 ± 2.6
Collagen+NSC23766 (300 μM)		2.9 ± 2

Blood was incubated with NSC23766 (300 μM) or solvent (water) in the presence of 2 mM RGDS for 5 min whilst stirring at 37°C before stimulation with TRAP or collagen. P-selectin expression was measured by flow cytometry. Values are mean ± SD, *n *= 3.

**Table 2:** Effect of NSC23766 and aspirin on P-selectin expression of PRP stimulated by ADP and collagen

Agonist	P-selectin expression (% positive cells)			
	**PRP**		**Aspirin-PRP**	
	
	**Control**	**Stimulated**	**Control**	**Stimulated**
		
ADP (5 μM)	1.4 ± 0.7	6 ± 2.8	1 ± 0.5	5.4 ± 2.6
ADP+NSC23766 (300 μM)	1.2 ± 1	1.8 ± 1.3	0.9 ± 0.4	2.1 ± 1.5
Collagen (5 μg/ml)	3.3 ± 3.1	42.4 ± 16.9	2 ± 1.3	6 ± 3.6
Collagen+NSC23766 (300 μM)	1.8 ± 1.3	3.1 ± 2.7	2 ± 1.5	2 ± 1.8

PRP or aspirin-pretreated PRP was incubated with NSC23766 (300 μM) or solvent (water) in the presence of 1 mM RGDS for 5 min whilst stirring at 37°C in the lumi-aggregometer before stimulation with ADP or collagen. P-selectin expression was measured by flow cytometry. Values are mean ± SD, *n *= 4.

### NSC23766 inhibits P-selectin expression and platelet aggregation stimulated by ADP independently of platelet cyclooxgenase activity

Aspirin reduced P-selectin expression of PRP by 89.8%, when stimulated with collagen but not when stimulated with ADP (Figure 3B). NSC23766 (300 μM) almost completely inhibited ADP-induced P-selectin expression in non-aspirin and aspirin-pretreated PRP (Table 2), and reduced ADP-stimulated platelet aggregation of untreated PRP and aspirin-pretreated PRP to a similar degree, by 70% and 75%, respectively (Figure 3B). NSC23766 (300 μM) also inhibited ADP-induced platelet aggregation in blood by 70% and 75% in the absence or presence of aspirin, respectively (Figure 3A). The results indicate that NSC23766 effectively inhibits α-granule secretion and platelet aggregation stimulated by ADP, and that the mechanism is independent of platelet prostaglandin-endoperoxide and thromboxane formation.

### NSC23766 inhibits human plaque-induced platelet thrombus formation under flow conditions

**Figure 4:**
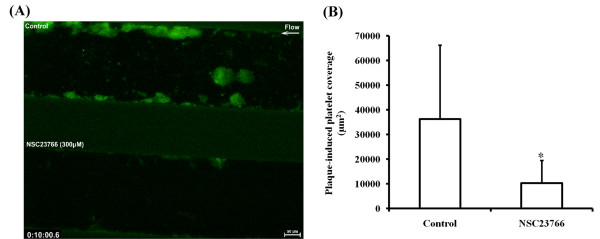
**Effect of NSC23766 on atherosclerotic plaque-induced platelet thrombus formation under arterial flow conditions**. Hirudin-anticoagulated blood pre-incubated with H_2_O or with NSC23766 (300 μM) for 5 min was perfused over plaque-coated surfaces for 10 min at 37°C at a shear rate of 1500 s^-1^. (A) representative flow images of control (upper channel) and NSC23766 treated blood (lower channel) 10 min after start of the flow; Platelets are visualized by mepacrine fluorescence; (B) bar diagram (values are mean ± SD; *n *= 5). * p < 0.002.

The effects of NSC23766 on human plaque-induced platelet aggregation and thrombus formation under arterial flow conditions are shown in Figure 4. After perfusion of hirudin-anticoagulated blood over plaque-coated surfaces at 37°C with a wall shear rate of 1500 s^-1^, rapid platelet adhesion and aggregate formation were observed (additional file 3 Movie S1; Figure 4a). The platelet coverage of the plaque-coated channels 10 min after start of flow was 36314 ± 30013 μm^2 ^(mean ± SD; *n *= 5). NSC23766 (300 μM) reduced plaque-induced platelet adhesion and aggregate formation. After NSC23766 incubation of blood, the platelet coverage was inhibited by 72% to 10322 ± 9226 μm^2 ^(mean ± SD; *n *= 5; *p *< 0.002).

## Discussion

In the present study, we have provided further evidence for a central role of Rac1 in the regulation of secretion and aggregation of human platelets activated by a broad range of platelet stimuli including atherosclerotic plaque. Moreover, we have demonstrated the efficacy of NSC23766 to inhibit platelet secretion and aggregation induced by these stimuli in blood, and we have shown that NSC23766 reduces plaque-induced platelet thrombus formation under arterial flow conditions.

Blood platelets are often studied after purifying platelets from their milieu, which excludes the influence exerted by other blood cells and factors present in plasma (e.g., high concentrations of albumin and fibrinogen, lipids exposed on LDL and HDL particles) on the physiological platelet response. Sometimes, pharmacological or physiological platelet inhibitors even fail to act on platelets in blood. For example, lysophosphatidic acid-receptor antagonists effective in washed platelets do not inhibit lysophosphatidic acid stimulation of platelets in PRP and blood (Rother E, Khandoga AL, Siess W, unpublished data), and PGI_2_, in contrast to washed platelets and PRP, was reported to be unable to inhibit platelet aggregation induced by arachidonic acid in whole blood [20]. Therefore, it was important to study the effect of NSC23766 on platelet activation in blood and PRP.

NSC23766 (300 μM) was able to almost completely block (~95% inhibition) platelet aggregation induced by TRAP (5 μM) in whole blood similar to thrombin- (0.5 U/ml) induced aggregation of washed platelets [13]. Thrombin activates PAR-1 and PAR-4 receptors, whereas TRAP only the PAR-1 receptor. A previous study has shown rapid activation and redistribution of Rac from the platelet interior to the cell periphery after TRAP-induced activation of platelets indicating that PAR-1 activation stimulates Rac [21]. It is not known whether PAR-4 activation also signals to Rac1 activation.

NSC23766 was also able to block human platelet aggregation in blood induced by other platelet agonists, such as fibrillar collagen, atherosclerotic plaque, and ADP, suggesting a central role of Rac1 signaling downstream of GPVI (collagen and atherosclerotic plaque) [5] and ADP receptors. These results are in part supported by studies of Rac1-deficient mice platelets, which showed inhibition of GPVI-dependent platelet activation [9,12,14]. However, in sharp contrast to two of these studies which reported only inhibition of collagen-stimulated, but not thrombin-induced platelet activation in Rac1-deficient mice [9,14], our study shows that Rac1 plays a role in platelet activation induced by all stimuli studied. Concerning the mechanism of ADP-receptor signaling to Rac in human platelets, it was shown that externally added ADP activates Rac through the activation of the P2Y_1 _receptor/G_q _pathway. However, when ADP was secreted from TRAP-stimulated platelets activation of the P2Y_12 _receptor/G_i _pathway played a central role [22].

Dose-response curves showed that NSC23766 inhibited human platelet aggregation in blood and PRP stimulated by all these agonists with a similar IC_50 _ranging between 50 to 70 μM. NSC23766 acts by disrupting the interaction of Rac1 with TrioN or Tiam1 Rac-GEFs, and it has been shown to inhibit *in vitro *both Rac1-TrioN binding and GEF activity of TrioN in a dose dependent manner, achieving 50% inhibition at 50 μM [15]. It is puzzling that the IC_50 _of NSC23766 for inhibition of stimulus-induced platelet aggregation in blood was found to be in the same range as the IC_50 _of NSC23766 in the *in vitro *reconstitution system consisting only of the two proteins Rac1 and TrioN. We expected that much higher concentrations of NSC23766 would be needed to inhibit Rac1 in platelets in blood considering the possible binding of the drug to plasma proteins and other blood cells and its crossing of the cell membrane before reaching its target Rac1 in the platelet interior. Platelet proteome data do not indicate the expression of TrioN or Tiam1 in human platelet (http://plateletweb.bioapps.biozentrum.uni-wuerzburg.de). One possible reason that μM concentrations of NSC23766 were effective in inhibiting Rac1 in platelets in blood is that other Rac1-GEFs might be present in human platelets which have a lower affinity to Rac1 than TrioN or Tiam1 and are thus displaced by lower (nM) drug concentrations *in vitro*.

Experiments using RGDS to block the integrin α_IIb_β_3 _showed that NSC23766 inhibited stimulus-induced secretion of dense granule as well as alpha granule contents in blood and PRP. These results indicate that NSC23766 also primarily inhibits platelet secretion and subsequently platelet aggregation in blood and PRP confirming previous studies in thrombin-stimulated washed platelet suspensions [12,13]. NSC23766 (300 μM) completely inhibited platelet P-selectin expression stimulated by collagen and TRAP in blood, but under the same experimental conditions (stirring, presence of RGDS), it did not inhibit completely ATP-secretion (inhibition of 73% after TRAP stimulation and of 85% after collagen stimulation). We reasoned that NSC23766 might be so effective in inhibiting collagen- and TRAP-induced platelet aggregation and platelet P-selectin expression in blood because it might inhibit the action of the residual secreted ADP on platelets. Indeed, NSC23766 inhibited ADP-induced aggregation by 70% and 75% in blood and PRP, respectively and completely in P-selectin expression.

Another important observation of our study concerns the role of integrin α_IIb_β_3 _outside-in signaling in the regulation of ATP-secretion in stirred activated blood. RGDS reduced ATP-secretion of stirred blood stimulated with collagen (0.5 μg/ml) and TRAP (5 μM) by 63% and 26%, respectively, indicating that integrin α_IIb_β_3 _signaling stimulated by platelet-to-platelet contact plays a role that is more important in collagen- than in TRAP-induced dense granule secretion of platelets in blood. These results are in line with a previous study of mice PRP showing the important role of the integrin  α_IIb_β_3 _in mediating secretion after stimulation with low level (2.5 μg/ml) collagen [23].

Aspirin, which reduced P-selectin expression of collagen-stimulated hirudin-anticoagulated PRP by 90%, was ineffective in inhibiting P-selectin expression when hirudin PRP was stimulated with ADP, confirming a previous study in citrated PRP [24]. Thus, aspirin fails to inhibit α-granule secretion after ADP stimulation of platelets independent of the anticoagulant used. The findings are in contrast to the results of dense granule secretion in citrated PRP, where aspirin is well known to inhibit dense granule secretion and the secondary wave of platelet aggregation after ADP stimulation [25]. Interestingly, we found that NSC23766 was equally effective in aspirin- and non-aspirin pretreated platelets in reducing P-selectin expression as well as platelet aggregation stimulated by ADP. Two conclusions can be drawn from these results: (1) NSC23766 is much more effective than aspirin in inhibiting the effect of ADP on platelets in blood and (2) NSC23766 inhibits α-granule secretion and platelet aggregation stimulated by ADP independent of platelet prostaglandin-endoperoxide and thromboxane formation.

## Conclusion

Our data clearly demonstrate the central role of Rac1 in secretion and subsequent platelet aggregation in blood upon activation by a wide array of platelet stimuli including atherosclerotic plaque. Rac1 inhibition by NSC23766 prevented platelet secretion from both α-granules and dense granules. We suggest that by inhibiting specifically platelet secretion, the pharmacological targeting of Rac1 could be an interesting approach in the development of future antiplatelet drugs.

## Competing interests

The authors declare that they have no competing interests.

## Authors' contributions

SD designed and performed the experiments, collected the results and analyzed the data. DP contributed by designing some of the experiments and interpreting the results. AKL participated in helping to perform the flow experiments. RB provided human plaque material. WS planned the study, assisted in designing the experiments, discussed and interpreted the results throughout the study, and wrote together with SD and DP the paper. All the authors have read and approved the final manuscript.

## Supplementary Material

Additional file 1 **Figure S1. Effect of NSC23766 on ATP-secretion and aggregation of PRP stimulated with collagen**. PRP was pre-incubated with or without 300 μM NSC23766 (for 5 min), with or without 1 mM RGDS (for 2 min; added 3 min after NSC23766 or H_2_O) whilst stirring at 37°C before stimulation with collagen (1.25 μg/ml). (A) Top, tracings of light transmission and ATP-secretion of PRP stimulated by collagen with or without NSC23766. Bottom, tracings of light transmission and ATP-secretion of PRP stimulated by collagen with or without NSC23766 in the presence of RGDS. (B) Dose-response curve of NSC23766 on platelet aggregation and ATP-secretion induced by collagen (1.25 μg/ml). Values are mean ± SD (*n *= 3). Click here for file

Additional file 2 **Figure S2. Effect of NSC23766 on ATP-secretion and aggregation of PRP stimulated with plaque**. PRP was pre-incubated with or without 300 μM NSC23766 (for 5 min), with or without 1 mM RGDS (for 2 min; added 3 min after NSC23766 or H_2_O) whilst stirring at 37°C before stimulation with plaque (0.62 mg/ml). (A) Top, tracings of light transmission and ATP-secretion of PRP stimulated by plaque with or without NSC23766. Bottom, tracings of light transmission and ATP-secretion of PRP stimulated by plaque with or without NSC23766 in the presence of RGDS. (B) Dose-response curve of NSC23766 on platelet aggregation and ATP-secretion induced by plaque (0.62 mg/ml). Values are mean ± SD (*n *= 3). Click here for file

Additional file 3 **Movie S1. Effect of NSC23766 on human plaque-induced platelet thrombus formation under arterial flow conditions**. Hirudin-anticoagulated blood was incubated with mepacrine to visualize platelets by fluorescence. Blood was perfused (direction right to left) over atherosclerotic plaque-coated microfluidic chambers and observed for 10 min. Upper channel, control; lower channel, blood pre-treated with 300 μM NSC23766. In the upper channel, rapid platelet adhesion and aggregate formation (green fluorescence) occurred, mainly at the edges of the channel, where also the majority of plaque material is present (as seen by phase contrast microscopy before start of the flow experiments). NSC23766 reduced platelet adhesion and aggregate formation. The video is in. mov format and can be viewed using Quick time player on different PCs with Windows XP or Vista. Click here for file
