# Supplementary material for: Rac1-mediated signaling plays a central role in secretion-dependent platelet aggregation in human blood stimulated by atherosclerotic plaque
Source: J Transl Med. 2010 Dec 6;8:128. doi: 10.1186/1479-5876-8-128 (PMC3018435; doi:10.1186/1479-5876-8-128)
Supplement: Additional file 2 — Figure S2. Effect of NSC23766 on ATP-secretion and aggregation of PRP stimulated with plaque. PRP was pre-incubated with or without 300 μM NSC23766 (for 5 min), with or without 1 mM RGDS (for 2 min; added 3 min after NSC23766 or H2O) whilst stirring at 37°C before stimulation with plaque (0.62 mg/ml). (A) Top, tracings of light transmission and ATP-secretion of PRP stimulated by plaque with or without NSC23766. Bottom, tracings of light transmission and ATP-secretion of PRP stimulated by plaque with or without NSC23766 in the presence of RGDS. (B) Dose-response curve of NSC23766 on platelet aggregation and ATP-secretion induced by plaque (0.62 mg/ml). Values are mean ± SD (n = 3). [file 1479-5876-8-128-S2.PDF]

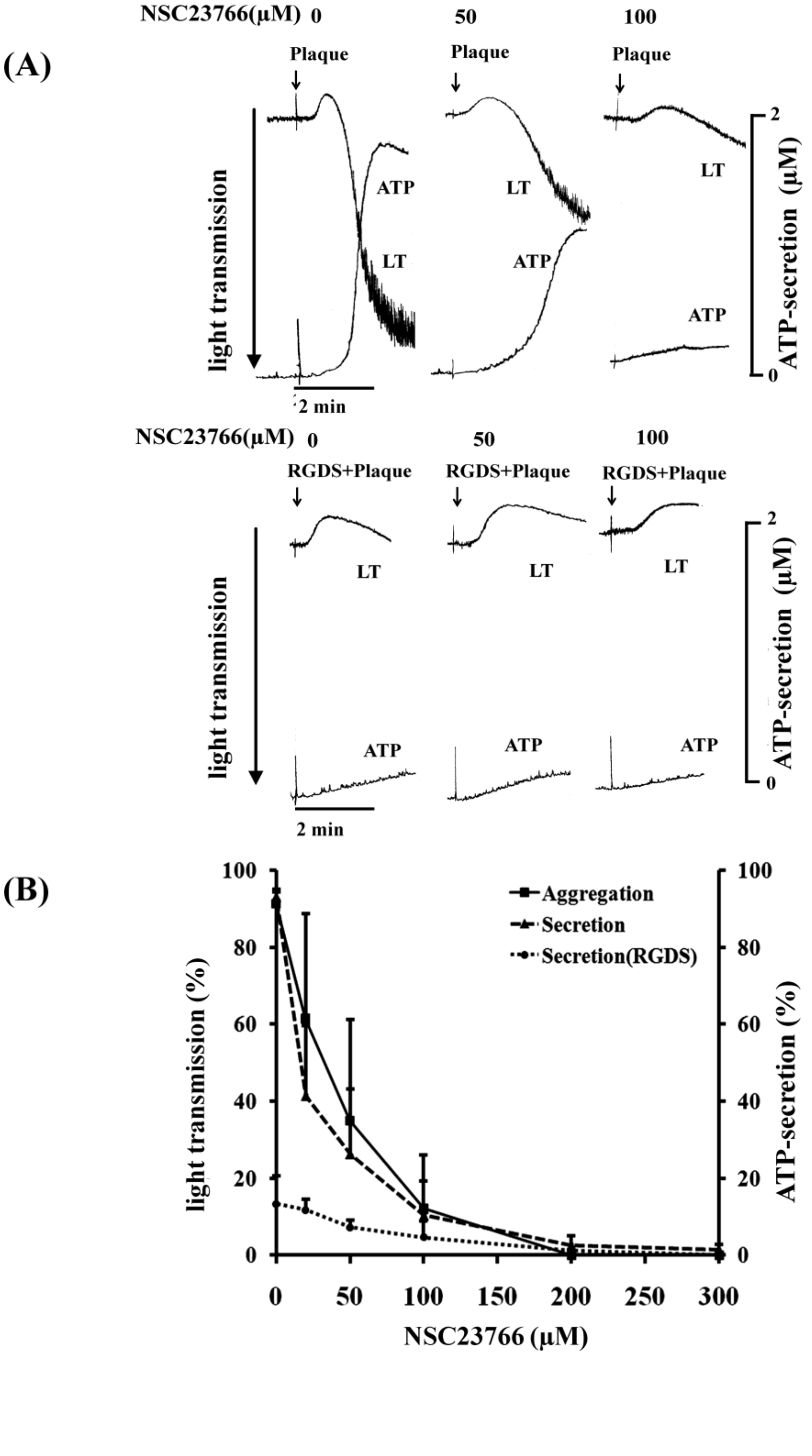
(A)
NSC23766(μM) 0
50
100
Plaque
light transmission
ATP
LT
2 min
ATP-secretion (μM)
2
0
RGDS+Plaque
(B)
Aggregation
Secretion
Secretion(RGDS)
light transmission (%)
ATP-secretion (%)
NSC23766 (μM)
0
50
100
150
200
250
300
20
40
60
80

Additional file 2

Figure S2

Effect of NSC23766 on ATP-secretion and aggregation of PRP stimulated with plaque

PRP was pre-incubated with or without 300 µM NSC23766 (for 5 min), with or without 1mM RGDS (for 2 min; added 3 min after NSC23766 or H_2O) whilst stirring at 37°C before stimulation with plaque (0.62mg/ml). (A) Top, tracings of light transmission and ATP-secretion of PRP stimulated by plaque with or without NSC23766. Bottom, tracings of light transmission and ATP-secretion of PRP stimulated by plaque with or without NSC23766 in the presence of RGDS. (B) Dose-response curve of NSC23766 on platelet aggregation and ATP-secretion induced by plaque (0.62mg/ml) .Values are mean ±SD (*n*=3).
